# Phylogenetic Analysis of Tanypodinae (Chironomidae, Diptera) Infered From Whole Mitochondrial Genomes

**DOI:** 10.1002/ece3.72975

**Published:** 2026-02-13

**Authors:** Wen‐Bin Liu, Jia‐Xin Nie, Ya‐Ning Tang, Zi‐Ming Shao, Cheng‐Yan Wang, Chun‐Cai Yan

**Affiliations:** ^1^ Tianjin Key Laboratory of Conservation and Utilization of Animal Diversity Tianjin Normal University Tianjin China

**Keywords:** evolution, mitochondrial genome, phylogenetic analysis, Tanypodinae

## Abstract

The family Chironomidae, particularly the subfamily Tanypodinae, represents a significant component of freshwater ecosystems. This investigation sought to clarify the evolutionary affinities within Tanypodinae by leveraging whole‐mitochondrial genome data. We generated the inaugural full mitogenomic sequences for 22 Tanypodinae taxa, added the mitochondrial genome sequence of another individual of *Thienemannimyia tripunctata*, merged these novel records with previously released chironomid mitochondrial genomes, and performed an integrated comparative mitogenomic survey encompassing 55 Tanypodinae species together with nine outgroup representatives from allied genera. The mitogenomes were characterized through de novo assembly, annotation, and comparative analysis. Our results demonstrated that the mitogenomes of Tanypodinae species exhibit a conserved structural organization, with the majority of genes arranged in the typical insect gene order. Phylogenies were reconstructed via Bayesian Inference (BI) and Maximum Likelihood (ML) analyses of the amino‐acid alignments of 13 protein‐coding genes (PCGs), corroborating the monophyly of tribe Pentaneurini and the sister‐group affinity between Procladiini and Tanypodini. These outcomes furnish novel insights into the evolutionary characteristics and phylogenetic framework of Tanypodinae while substantially expanding the mitogenomic archive for Chironomidae.

## Graphical abstract

We generated the inaugural full mitogenomic sequences for 22 Tanypodinae taxa, and the mitochondrial genome sequence of another individual of Thienemannimyia tripunctata has been added; merged these novel records with previously released chironomid mitochondrial genomes; and performed an integrated comparative mitogenomic survey encompassing 55 Tanypodinae species together with nine outgroup representatives from allied genera.

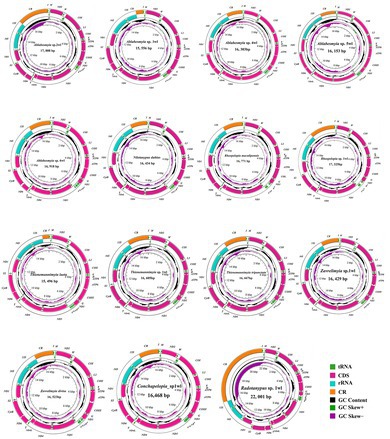

## 1 Introduction

Chironomidae, positioned within the Diptera order, stands as the most broadly dispersed and frequently the most abundant insect lineage in freshwater ecosystems, with occurrences further confirmed in terrestrial and marine habitats (Armitage et al. 1995). The remarkable species diversity of this family can be traced back to its ancient origins, relatively low vagility that promotes isolation, and strong evolutionary plasticity (Andersen et al. 2013; Pinder 1989). Many species within the family exhibit short life cycles and high population densities, which have furnished valuable foundational data regarding ecosystem productivity and population dynamics (Bello‐González et al. 2024; Liu et al. 2022).

Tanypodinae, ranking third in species richness among the subfamilies of Chironomidae, is distributed across nearly all continents except Antarctica and encompasses 57 genera assigned to nine currently recognized tribes (Krosch et al. 2021). The diagnostic characteristics of the Tanypodinae include the position of crossvein MCu relative to FCu (either before or beyond it), the specific shape of the tibial spurs, and the inward or forward orientation of the gonostyles (Murray and Fittkau 1989). The majority of Tanypodinae larvae are generally considered carnivorous and are primarily found in various lotic and lentic water habitats, as well as occasionally in moist semi‐terrestrial environments; however, most species tend to prefer standing water or slow‐flowing river sections, showing an adaptation to warmer waters that is reflected in their global distribution, with their diversity decreasing in higher altitudes or cooler regions (Andersen et al. 2013; Ashe et al. 1987).

While phylogenetic analyses that integrate both morphological and molecular data consistently support the monophyly of Tanypodinae, the relationships within the subfamily have been found to be inconsistent and with variable levels of support (Cranston et al. 2012; Gao et al. 2024; Krosch et al. 2017, 2021; Sæther 1977; Silva and Ekrem 2016). Within the subfamily Tanypodinae, there are two major lineages: the Pentaneurini Hennig, 1950, which includes most species, and a group of eight tribes, among which Procladiini and Tanypodini are sister taxa forming terminal lineages that may even need to be merged into a single tribe (Krosch et al. 2017, 2021; Xiao et al. 2025). The placement of these three tribes within the subfamily Tanypodinae and their inter‐relationships warrant further investigation.

Insect mitochondrial genomes serve as powerful molecular markers for Diptera identification and phylogenetic reconstruction owing to their characteristics: they are compact, maternally transmitted, and structurally stable, and they exhibit rare recombination and accelerated evolutionary rates. Chironomidae research is a prime beneficiary of these attributes (Cameron 2014; Liu et al. 2025, 2024; Song et al. 2019; Xiao et al. 2025). This study reports the first complete mitogenomes for 22 Tanypodinae species, and the mitochondrial genome sequence of another individual of *Thienemannimyia tripunctata* has been added, enhancing Chironomidae's mitogenomic resources. To extend the scope of our investigation, an additional 23 publicly available mitogenomes were integrated into the dataset, allowing an in‐depth characterization of these mitochondrial genomes. Leveraging diverse sequence repositories, we inferred the phylogenetic affinities among genera within the subfamily Tanypodinae by implementing both Bayesian Inference (BI) and Maximum Likelihood (ML) frameworks, ensuring analytical robustness and scientific rigor. Our research has confirmed the monophyly of tribe Pentaneurini, and that tribes Procladiini and Tanypodini are sister clades.

## 2 Materials and Methods

### 2.1 Specimen Acquisition and Sequencing

This study reports the first analysis included five species of *Ablabesmyia*, seven species of *Procladius*, one species of *Radotanypus*, nine species of Pentaneurini tribe, and one species of *Tanypus*. All specimens were collected exclusively within China. Permission was obtained for the collection of all insect specimens used in this study. Sample collection was conducted in accordance with the Wildlife Protection Law of the People's Republic of China and was approved by the following national nature reserves: Huaping in Guangxi, Qizimeishan in Xuanen County of Hubei, Gutianshan in Kaihua County of Zhejiang, and Jiuzhaigou in Sichuan. The work was carried out with permission from the National Nature Reserves Administration of China. All sampling activities were also authorized under a broader scientific collection permit issued by the National Forestry and Grassland Administration of China, which supports university‐led scientific surveys within protected areas across the country. Comprehensive specimen details are provided in Table 1.

**TABLE 1:** Collection information of newly sequenced species in this study.

Species	Sample ID	Location	Longitude and latitude	Date	Collector
*Ablabesmyia* sp. 2wl	SHP001	Huaping National Nature Reserve, Guangxi Zhuang Autonomous Region, China	109°58′10″ E, 25°39′15″ N	22 July 2021	Yao Yuan
*Ablabesmyia* sp. 3wl	XBZ169	Xishuangbanna Tropical Botanical Garden, Xishuangbanna Dai Autonomous Prefecture, Yunnan Province, China	101°25′00″ E, 21°41′00″ N	19 April 2014	Wan Qiang
*Ablabesmyia* sp. 4wl	XBZ168	Xishuangbanna Tropical Botanical Garden, Xishuangbanna Dai Autonomous Prefecture, Yunnan Province, China	101°25′00″ E, 21°41′00″ N	22 April 2014	Wan Qiang
*Ablabesmyia* sp. 5wl	TES002	Qizimei Mountain National Nature Reserve, Xuan'en County, Enshi Tujia and Miao Autonomous Prefecture, Hubei Province, China	109°38′30″ E, 29°39′30″ N	9 July 2015	Sun Bingjiao
*Ablabesmyia* sp. 6wl	KTS455	Gutianshan National Nature Reserve, Kaihua County, Quzhou City, Zhejiang Province, China	118°03′56″ E, 29°10′32″ N	8 July 2017	Zhao Guangjun
*Conchapelopia* sp. 1wl	3XB002	Beichuan Wetland Park, Chengzhong District, Xining City, Qinghai Province, China	101°49′17″ E, 36°34′03″ N	12 November 2023	Gao Xin
*Nilotanypus dubius*	VEH001	Baoguo Temple, Emeishan City, Leshan City, Sichuan Province, China	103°44′33.6″ E, 29°33′22.3″ N	22 July 2015	Liu Wenbin
*Procladius choreus*	KSJ130	Shangjie Village, Furong Town, Yueqing City, Zhejiang Province, China	111°18′57.64″ E, 37°35′40.52″ N	20 May 2014	Liu Wenbin
*Procladius nigriventris*	TES003	Qizimei Mountain National Nature Reserve, Xuan'en County, Enshi Tujia and Miao Autonomous Prefecture, Hubei Province, China	109°38′30″ E, 29°39′30″ N	10 July 2015	Sun Bingjiao
*Procladius paludicola*	SHC002	Xiaoyun'an Town, Luocheng County, Hechi City, Guangxi Zhuang Autonomous Region, China	109°04′82″ E, 24°88′24″ N	11 April 2015	Liu Wenbin
*Procladius* sp. 1wl	YNN001	Nujiang River, Nagqu City, Tibet Autonomous Region, China	98°67′19″ E, 27°91′25″ N	15 June 2023	Zhao Lin
*Procladius* sp. 2wl	3XB001	Beichuan Wetland Park, Chengzhong District, Xining City, Qinghai Province, China	101°49′17″ E, 36°34′03″ N	12 November 2023	Gao Xin
*Procladius* sp. 3wl	VJZ066	Jiuzhaigou National Park, Jiuzhaigou County, Ngawa Tibetan and Qiang Autonomous Prefecture, Sichuan Province, China	103°54′00″ E, 33°12′00″ N	15 July 2019	Ge Xinyu
*Procladius* sp. 4wl	FBX001	Benxi City, Liaoning Province, China	123°90′90″ E, 40°87′32″ N	24 July 2023	Song Chao
*Radotanypus* sp. 1wl	YNN002	Nujiang River, Nagqu City, Tibet Autonomous Region, China	98°67′19″ E, 27°91′25″ N	16 June 2023	Zhao Lin
*Rheopelopia maculipennis*	KLT191	Lin'an District, Hangzhou City, Zhejiang Province, China	118°51′00″ E, 29°56′00″ N	29 July 2011	Lin Xiaolong
*Rheopelopia* sp. 1wl	LHS001	Huangshan National Forest Park, Huangshan City, Anhui Province, China	117°57′00″ E, 30°25′00″ N	13 May 2015	Wan Qiang
*Tanypus* sp. 1wl	WST015	Songtao Miao Autonomous County, Tongren City, Guizhou Province, China	109°11′53″ E, 28°9′31″ N	29 July 2021	Cao wei
*Thienemannimyia laeta*	KTM381	Tianmu Mountain, Lin'an District, Hangzhou City, Zhejiang Province, China	119°25′00″ E, 30°20′00″ N	10 April 2024	Song Chao
*Thienemannimyia* sp. 1wl	AFS001	Qinglong Lake, Fangshan District, Beijing Municipality, China	116°03′96″ E, 39°77′73″ N	21 March 2024	Wang Chengyan
*Thienemannimyia tripunctata*	VJZ067	Jiuzhaigou National Park, Jiuzhaigou County, Ngawa Tibetan and Qiang Autonomous Prefecture, Sichuan Province, China	103°54′00″ E,33°12′00″ N	15 July 2019	Ge Xinyu
*Zavrelimyia* sp. 1wl	KNH001	Fenghua River, Haishu District, Ningbo City, Zhejiang Province, China	121°40′78″ E, 29°67′12″ N	14 May 2015	Qi Xin
*Zavrelimyia divisa*	KSJ129	Shangjie Village, Furong Town, Yueqing City, Zhejiang Province, China	111°18′58″ E, 37°35′41″ N	17 July 2011	Lin Xiaolong

A total of 64 species were analyzed. The ingroup encompassed 55 species: 32 with complete mitochondrial genomes downloaded from GenBank, plus 23 whose mitogenomes are presented here de novo, and the newly added mitochondrial genome of *Thienemannimyia tripunctata* (Jiang et al. 2022; Lin et al. 2022; Xiao et al. 2025; Liu et al. 2025). The outgroup consisted of nine species whose mitogenomic data were also obtained from GenBank (Lin et al. 2022). All were drawn from closely allied genera. Further particulars are provided in Table 2.

**TABLE 2:** Sequences used in this study.

Species	GenBank accession	References
*Ablabesmyia longistyla*	*PQ323369*	Liu et al. 2025
*Ablabesmyia monilis*	*PQ323370*	Liu et al. 2025
*Ablabesmyia monilis*	*OP006242*	Xiao et al. 2025
*Ablabesmyia prorasha*	*PQ323371*	Liu et al. 2025
*Ablabesmyia prorasha*	*OP006228*	Xiao et al. 2025
*Ablabesmyia rhamphe*	*PQ323372*	Liu et al. 2025
*Ablabesmyia* sp. 15XL	*PQ323378*	Liu et al. 2025
*Ablabesmyia* sp. 1wl	*PQ323375*	Liu et al. 2025
*Ablabesmyia* sp. 2wl	*PV962260*	This study
*Ablabesmyia* sp. 3wl	*PV962261*	This study
*Ablabesmyia* sp. 3XL	*PQ323376*	Liu et al. 2025
*Ablabesmyia* sp. 4wl	*PV962262*	This study
*Ablabesmyia* sp. 5wl	*PV962263*	This study
*Ablabesmyia* sp. 6wl	*PV962264*	This study
*Ablabesmyia* sp. 7XL	*PQ323377*	Liu et al. 2025
*Clinotanypus yani*	*MW373524*	Lin et al. 2022
*Conchapelopia* sp. 1wl	*Pending*	This study
*Conchapelopia togamaculosa*	*PQ323373*	Liu et al. 2025
*Conchapelopia togamaculosa*	*OP006233*	Xiao et al. 2025
*Denopelopia irioquerea*	*PQ323374*	Liu et al. 2025
*Denopelopia bractea*	*OP006240*	Xiao et al. 2025
*Djalmabatista sinica*	*OP006241*	Xiao et al. 2025
*Larsia myagsensis*	*OP006230*	Xiao et al. 2025
*Macropelopia paranebulosa*	*OP006236*	Xiao et al. 2025
*Natarsia qinlingica*	*OP006229*	Xiao et al. 2025
*Nilotanypus dubius*	*PX057726*	This study
*Psectrotanypus dyari*	*OP006244*	Xiao et al. 2025
*Procladius longistilus*	*OP006232*	Xiao et al. 2025
*Procladius choreus*	*PV983390*	This study
*Procladius imicola*	*PV983385*	This study
*Procladius paludicola*	*PV983391*	This study
*Procladius* sp. 1wl	*PV983387*	This study
*Procladius* sp. 2wl	*PV983388*	This study
*Procladius* sp. 3wl	*PV983389*	This study
*Procladius* sp. 4wl	*PV983386*	This study
*Radotanypus* sp. 1wl	*PX057727*	This study
*Rheopelopia maculipennis*	*PX057728*	This study
*Rheopelopia* sp. 1wl	*PX057729*	This study
*Saetheromyia tedoriprima*	*OP006243*	Xiao et al. 2025
*Tanypus chinensis*	*OP006227*	Xiao et al. 2025
*Tanypus kraatzi*	*OP006235*	Xiao et al. 2025
*Thienemannimyia fuscipes*	*OP006231*	Xiao et al. 2025
*Thienemannimyia tripunctata*	*OP006237*	Xiao et al. 2025
*Trissopelopia* sp. 1XL	*OP006226*	Xiao et al. 2025
*Tanypus chinensis*	*OP006227*	Xiao et al. 2025
*Tanypus kraatzi*	*OP006235*	Xiao et al. 2025
*Tanypus punctipennis*	*MZ745054*	Jiang et al. 2022
*Tanypus* sp. 1wl	*PX057730*	This study
*Thienemannimyia laeta*	*PX057731*	This study
*Thienemannimyia* sp.	*PQ323379*	Liu et al. 2025
*Thienemannimyia* sp. 1wl	*PX057732*	This study
*Thienemannimyia tripunctata*	*PX057733*	This study
*Zavrelimyia dolosa*	*OP006239*	Xiao et al. 2025
*Zavrelimyia divisa*	*PX057734*	This study
*Zavrelimyia* sp. 1wl	*PX057735*	This study
*Cricotopus dentatus*	*OP006251*	Li et al. 2023
*Cricotopus bicinctus*	*OP006255*	Li et al. 2023
*Boreoheptagyia alulasetosa*	*MZ043574*	Lin et al. 2022
*Boreoheptagyia kurobebrevis*	*MZ043576*	Lin et al. 2022
*Monodiamesa* sp. *1*	*MW837769*	Lin et al. 2021
*Monodiamesa* sp. *2*	*MW837770*	Lin et al. 2021
*Chironomus transvaalensis*	*ON975023*	Li et al. 2022
*Chironomus circumdatus*	*ON975024*	Li et al. 2022
*Parochlus steinenii*	*NC027591*	Shin et al. 2023

Prior to genomic DNA isolation and morphological inspection, every specimen was immersed in 85%–95% ethanol and maintained at −20°C to ensure structural and molecular integrity.

### 2.2 Assembly, Annotation, and Composition Analyses

Mitogenomes assembly was performed de novo using NOVOPlasty v3.8.3 (Dierckxsens et al. 2017), with the *COI* barcode sequence serving as the initial seed. To enhance assembly optimization, we systematically evaluated multiple k‐mer lengths spanning 23–39 bp, employing a comparative approach to refine the mitogenome assembly parameters (Dierckxsens et al. 2017). Mitochondrial genome annotation followed the protocol described by Zheng et al. (2020) with modifications. Specifically, tRNA secondary structures were determined using the MITOS WebServer, whereas rRNAs and protein‐coding genes (PCGs) were manually annotated in Geneious. Sequence alignments were performed using the Clustal Omega algorithm (Kumar et al. 2016). The boundaries between PCGs and rRNAs were subsequently manually verified through alignment with reference sequences. Moreover, the synonymous (Ks) and non‐synonymous substitution rates (Ka) of PCGs were computed using DnaSP v6.0 (Rozas et al. 2017). The processed PCGs file was imported into the software, and the “mtDNA Drosophila” genetic code was selected for subsequent analysis. MEGA X was utilized to quantify relative synonymous codon usage, nucleotide constitution, and codon preferences across the mitogenomes. Compositional asymmetry and gene‐wise patterns were evaluated with SeqKit v0.16.0, a bioinformatics utility developed in Chongqing, China (Shen et al. 2016). Circular genome diagrams were rendered via the CGView Server (https://cgview.ca/; accessed 8 May 2025) (Rozas et al. 2017). Strand‐specific nucleotide biases were assessed by computing AT‐skew [(A − T)/(A + T)] and GC‐skew [(G − C)/(G + C)], thereby illuminating the evolutionary dynamics of the mitogenome.

### 2.3 Phylogenetic Analyses

To resolve phylogenetic affinities, we assembled a dataset comprising two ribosomal RNA (rRNA) loci and 13 PCGs retrieved from 50 complete mitochondrial genomes (Table 2). Both nucleotide and corresponding amino‐acid sequences were aligned with MAFFT v7.470 employing the L‐INS‐I strategy to ensure high fidelity (Katoh and Standley 2013). Handling of DNA Indels chose 5th state. Subsequent sequence trimming was executed with Trimal v1.4.1 (Barcelona, Spain) to prepare the aligned datasets for downstream phylogenetic analyses. This investigation relied on five discrete data partitions generated by FASconCAT‐G v1.04, each tailored as follows: (1) cds_fna, containing every codon position of the 13 PCGs; (2) cds12_rna, merging the first and second codon sites of the 13 PCGs with the two rRNA loci; (3) cds_rna, uniting all codon positions of the 13 PCGs and the two rRNA loci; (4) cds_faa, employing the deduced amino‐acid sequences of the 13 PCGs; and (5) cds12, restricted to the first and second codon sites of the 13 PCGs. To gauge between‐matrix heterogeneity, we utilized AliGROOVE v1.06 (Bonn, Germany), adopting the conceptual framework established in investigations (Capella‐Gutiérrez et al. 2009; Katoh and Standley 2013; Kück et al. 2014; Tamura et al. 2021). Amino Acid Substitution Matrix chose BLOSUM26. For ML analysis, best‐fit substitution models were implemented across individual gene partitions. Bootstrap resampling and node confidence assessment were executed with 1000 replicates to evaluate statistical reliability. Subsequent phylogenetic reconstruction employed IQ‐TREE v2.2.0.8 for ML tree inference and Phylobayes‐MPI v1.9 for Bayesian analysis, collectively providing comprehensive insights into the evolutionary dynamics among the examined mitochondrial genomes (Hoang et al. 2018; Kalyaanamoorthy et al. 2017; Lartillot et al. 2013; Minh et al. 2020).

## 3 Results and Interpretation

### 3.1 Mitogenomic Organization

**FIGURE 1:**
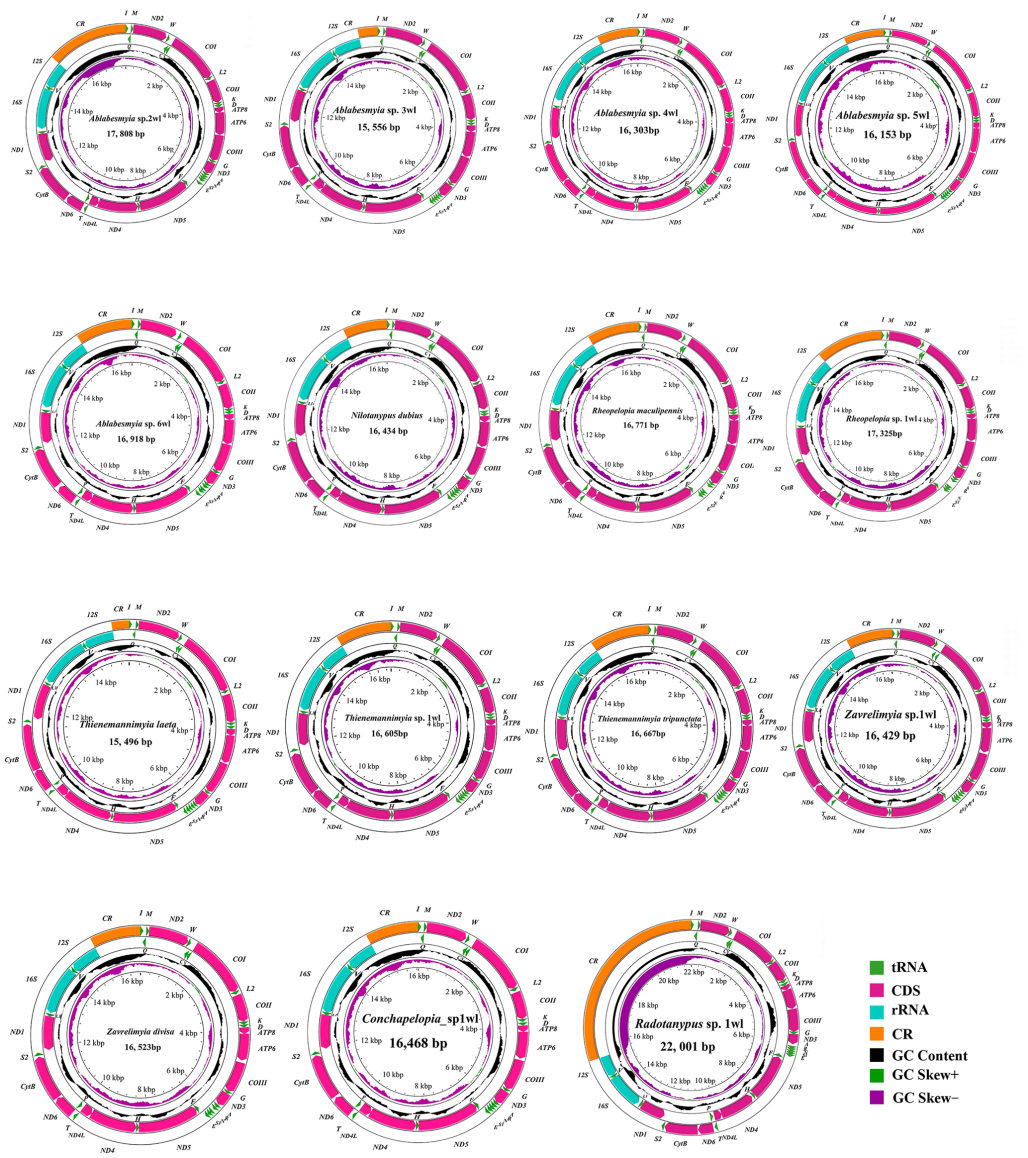
The mitochondrial genome map of diverse representative species across 15 species of Tanypodinae is presented, emphasizing their unique genomic features. PCGs and rRNAs are annotated with canonical abbreviations, whereas transfer RNAs (tRNAs) are abbreviated to single‐letter symbols to preserve clarity. An additional concentric ring depicts the mitogenome‐wide GC content, elucidating global trends in nucleotide composition. A subsequent ring presents GC‐skew indices, thereby extending the evaluation of strand‐specific base imbalance. The centermost ring reports the aggregate mitogenome length, integrating pivotal architectural metrics into a cohesive graphical summary.

**FIGURE 2:**
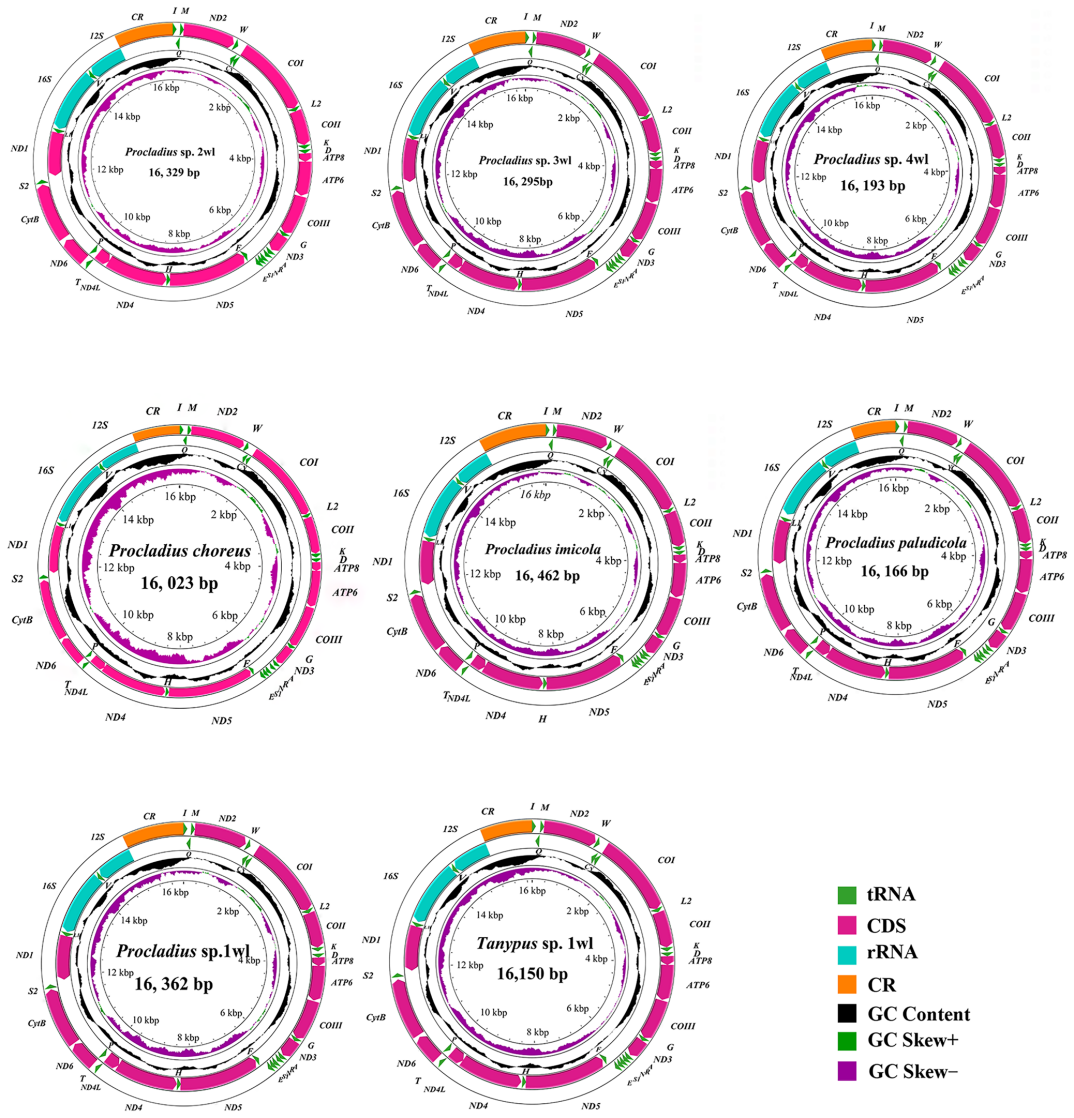
The mitochondrial genome map of diverse representative species across eight species of Tanypodinae is presented, emphasizing their unique genomic features. Detailed interpretation of the mitochondrial circular map refers specifically to the annotations provided in Figure 1.

The freshly obtained sequences spanned 15,496 base pairs in *Thienemannimyia laeta* to 22,001 bp in *Radotanypus* sp. 1wl. This variability chiefly stems from the variable length of the CR, which measures 421 bp in *Thienemannimyia laeta* and expands to 6623 bp in *Radotanypus* sp. 1wl (Table S1). All de novo mitogenome assemblies conform to the canonical architecture, comprising one CR and 37 genes—specifically 13 PCGs, 22 tRNAs, and 2 rRNAs—as depicted in Figures 1 and 2. The majority of assemblies align closely in size with previously documented Chironomidae mitogenomes. The salient sequence attributes of the examined taxa are graphically consolidated in Figures 1 and 2.

**FIGURE 3:**
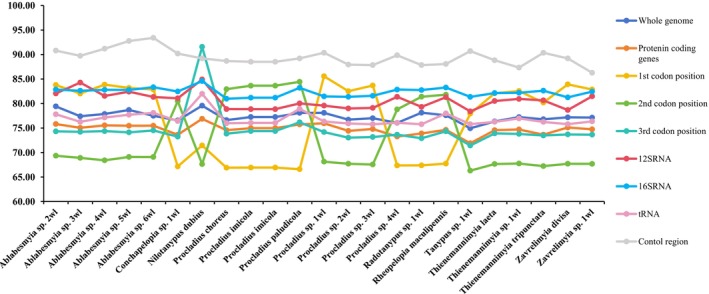
Difference in AT composition of the protein‐coding genes within Tanypodinae mitogenomes.

The nucleotide patterns of the recently assembled mitogenomes exhibited uniform trends across the analyzed specimens, as outlined in Table S1, mirroring the typical AT‐enriched inclination prevalent in Chironomidae and other insect lineages. Notable disparity emerged in the AT proportion of the mitochondrial genomes, spanning from 74.95% in *Tanypus* sp. 1wl to 79.57% in *Nilotanypus dubius* (Figure 3 and Table S1).

Strikingly, the CR exhibited the most pronounced AT enrichment, spanning 76.65% in *Conchapelopia togamaculosa* to 93.41% in *Ablabesmyia* sp. 3wl. By contrast, the AT proportion in tRNAs and PCGs was markedly lower than that observed in rRNAs (Table S1). All freshly reconstructed mitogenomes exhibited a negative GC‐skew, indicating a surplus of cytosine, whereas most displayed a positive AT‐skew, evidencing the predominance of adenine and thymine. GC‐skew spanned from −0.34 in *Radotanypus* sp. 1wl to −0.120 in *Procladius* sp. 1wl. AT‐skew varied between 0 in *Ablabesmyia* sp. 6wl and 0.05 in *Rheopelopia maculipennis*, with a single outlier *Radotanypus* sp. 1wl—showing an AT‐skew of −0.04. GC content itself fluctuated from 20.34% in *Ablabesmyia* sp. 2wl to 25.05% in *Tanypus* sp. 1wl, furnishing further insight into the nucleotide landscape of these mitogenomes (Table S1).

### 3.2 Protein‐Coding Genes, Codon Usage, and Evolutionary Rates

Conservation of gene length was observed across tRNA, PCGs, and rRNA in all examined species. Specifically, the cumulative length of the 13 PCGs in newly sequenced mitogenomes displayed minimal variation (11,210–11,239 bp). Comparative analysis with existing Chironomidae datasets revealed distinct codon position biases in the Pentaneurini tribe: *Ablabesmyia* and *Thienemannimyia* exhibited significantly elevated AT content at first codon positions relative to second and third positions in PCGs. In *Conchapelopia* and *Rheopelopia*, the AT proportion at the second codon sites of PCGs markedly exceeds that at the first and third sites. *Nilotanypus dubius* exhibits an opposite trend, with the third codon sites of PCGs displaying the highest AT proportion relative to the first and second sites. Within the Procladiini tribe, *Procladius* sp. 1wl, *Procladius* sp. 2wl, and *Procladius* sp. 3wl show elevated AT proportions at the first codon sites of PCGs, whereas the remaining taxa exhibit a pronounced increase at the second codon sites. Within the Tanypodini tribe, first codon positions in PCGs exhibited predominant AT content relative to second and third positions. Conversely, second codon positions demonstrated elevated AT content compared to first and third positions in the Macropelopiini tribe.

Strikingly, across the complete set of 55 mitogenomes, the majority exhibited a negative GC skew at the whole genome scale. Moreover, a negative AT skew was observed within the PCGs, spanning from −0.19 in *Ablabesmyia longistyla* to −0.17 in *Nilotanypus dubius*. The AT proportion fluctuated between 71.89% in *Tanypus* sp. 1wl and 76.88% in *Nilotanypus dubius*, whereas the GC content ranged from 23.12% in *Nilotanypus dubius* to 28.11% in *Tanypus* sp. 1wl (consult Table 2 for comprehensive details).

**FIGURE 4:**
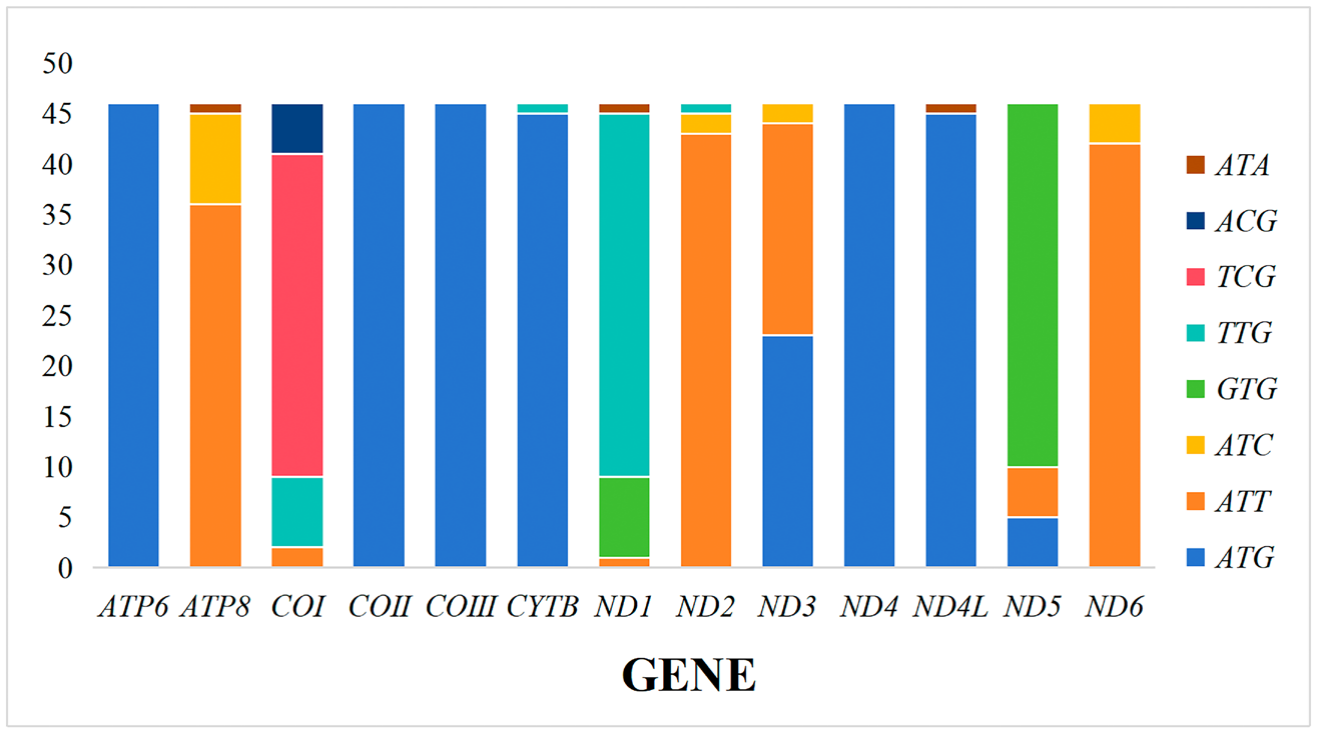
Start codons of protein‐coding genes among Tanypodinae mitogenomes.

13 PCGs of all species in the analyzed mitogenomes predominantly employed the canonical ATN start codon, consistent with typical insect mitochondrial initiation patterns. However, non‐canonical start codons were identified in specific genes. Most notably, the *COI* gene initiated with TCG in 32 species, TTG in 7 species, ACG in 5 species, and ATT in 2 species. The *ATP8* gene began with ATT in 36 species and ATC in nine species, while the *ATP6* gene started with ATG in all species. The *ND1* gene utilized TTG as its start codon in 36 species, GTG in eight species, and ATT in one species. Similarly, the *ND2* gene started with ATT in 43 species, ATC in two species, and TTG in one species; the *ND3* gene began with ATT in 21 species, ATG in 23 species, and ATC in 2 species. Furthermore, the *COII*, *COIII*, and *ND4* genes consistently started with ATG, the *CYTB* and *ND4L* genes with ATG in 45 species. The *ND5* gene started with GTG in 36 species, ATT in 5 species, and ATG in 5 species, while the *ND6* gene exclusively began with ATT in 42 species and ATC in four species (Figure 4).

Regarding termination codons, the predominant signal among the 13 PCGs was TAA. Deviations include *COI* and *COII*, which predominantly terminate with a solitary thymine (T) as an incomplete stop; *ND5*, exhibiting 29 instances of T and 32 of TAA; *ND6*, presenting two T and one TAG; *ND1*, characterized by seven TAG; and *ND3*, marked by four TAG codons.

**FIGURE 5:**
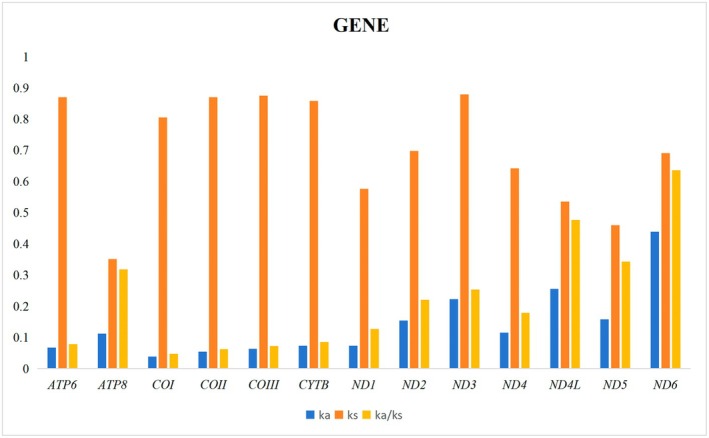
Evolutionary tempo of the 13 PCGs across Tanypodinae mitogenomes. The x‐axis enumerates the 13 PCGs, while the y‐axis indicates the Ka/Ks ratios.

The Ka/Ks ratio (ω), a well‐established indicator of sequence evolutionary tempo under selective pressure, remained uniformly below unity across all 13 PCGs. This pattern aligns closely with observations in other insect lineages, with a range spanning from 0.043 for *COI* to 0.891 for *ND2* (Figure 4). These PCGs can be ranked according to their evolutionary rates as follows: *ND2* > *ATP8* > *ND6* > *ND5* > *ND4* > *ND4L* > *ND3* > *ATP6* > *ND1 > CYTB > COIII > COII > COI*. Our results notably highlight that a substantial proportion of these genes have undergone evolutionary refinement under purifying selection, which functions to eliminate deleterious mutations and is modulated by heterogeneous selective forces. Notably, the diminished ω ratios observed in *COII* and *COI* genes signify a stringent selective environment, indicating the presence of robust evolutionary constraints. Conversely, the elevated ω values detected in *ND2*, *ATP8*, and *ND6* suggest comparatively relaxed purifying selection pressures, implying that these genetic elements may experience greater evolutionary latitude (Figure 5). These insights substantially enhance our grasp of the selective forces operating on these PCGs and highlight the central influence of natural selection in directing their patterns of sequence divergence.

The 55 mitochondrial tRNAs displayed pronounced length heterogeneity, spanning 1474–2153 base pairs (bp). Their AT fraction also diverged markedly, ranging from 75.71% in *Clinotanypus yani* to 81.99% in *Nilotanypus dubius*. Remarkably, nine tRNAs exhibited a negative AT‐skew, varying from −0.05 to −0.01. Conversely, GC content fluctuated between 18.01% in *Nilotanypus dubius* and 24.30% in *Clinotanypus yani*. Moreover, GC‐skew revealed substantial variability, extending from 0.12 in *Procladius paludicola* to 0.28 in *Thienemannimyia tripunctata*. Collectively, these findings underscore extensive nucleotide compositional and skew divergence across mitochondrial tRNAs within the examined taxa.

The rRNA segments displayed pronounced length heterogeneity, spanning 2136 bp in *Ablabesmyia* sp. 4wl to 2212 bp in *Nilotanypus dubius*. AT fractions remained uniformly elevated across all mitogenomes, fluctuating between 76.25% and 84.72%, while GC levels mirrored this variability, ranging from 15.28% to 23.74%. Although the majority of mitogenomes exhibited a negative AT‐skew between −0.10 and −0.01, *Thienemannimyia tripunctata* presented an AT‐skew of 0. A comprehensive compilation of these metrics is provided in Table S1.

### 3.3 Phylogenetic Relationships

**FIGURE 6:**
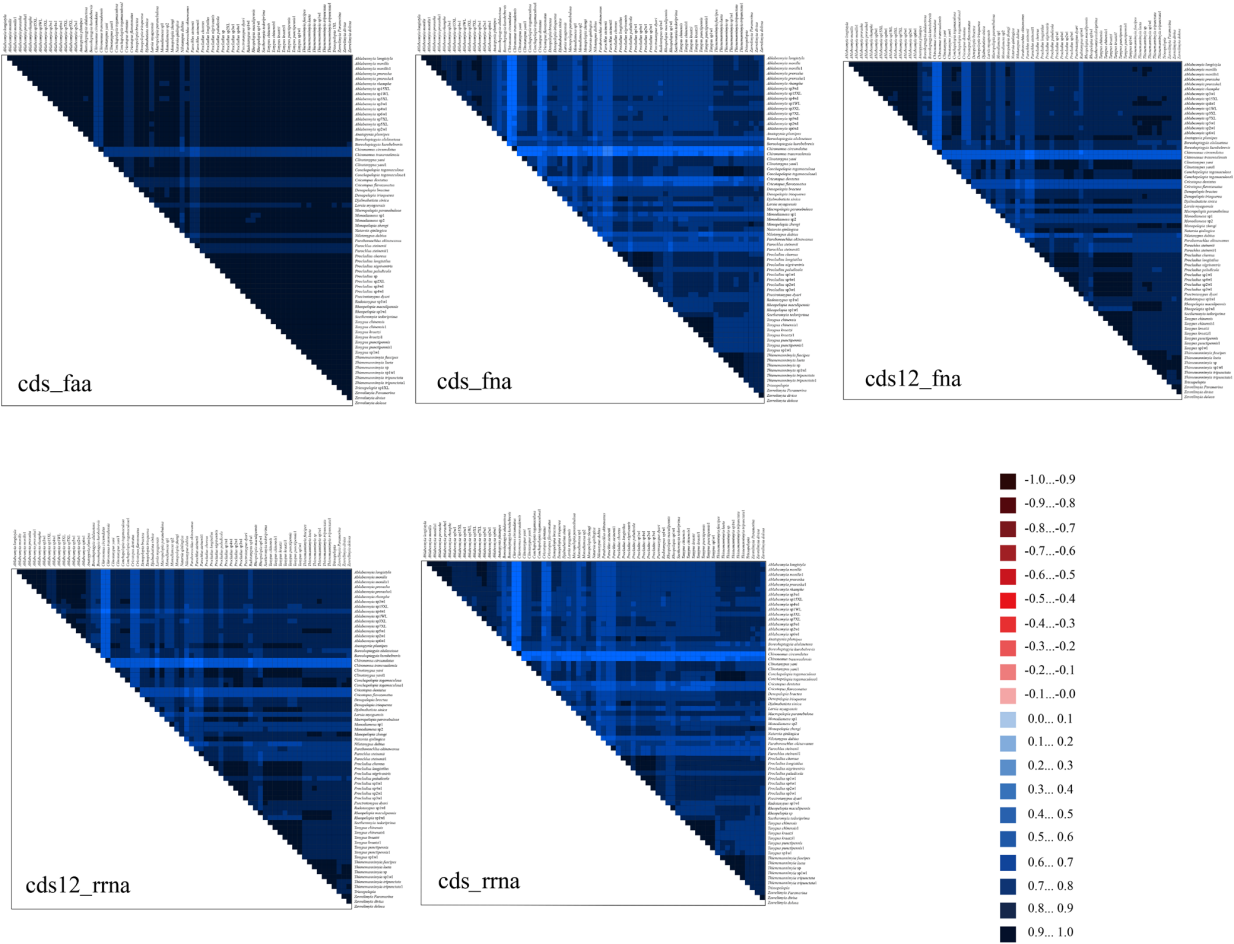
Genomic divergence across 55 Tanypodinae species was evaluated by examining their PCGs, amino‐acid sequences, and rRNAs. Homology patterns were visualized through color‐graded tiles calculated from AliGROOVE scores, extending from −1 (crimson, indicating pronounced divergence) to +1 (azure, denoting strong conservation). Within each tile, paler tones correspond to heightened sequence disparity, whereas deeper shades reflect diminished variability.

The examination of sequence divergence patterns elucidates the degree of mitochondrial gene sequence conservation across multiple taxa. Notably, due to the degeneracy of the genetic code, the amino acid (AA) dataset demonstrated minimal sequence divergence, whereas the combined coding and ribosomal RNA (cds12_rrna) dataset exhibited relatively elevated divergence levels (Figure 6). This observation suggests that the third codon positions in PCGs accumulate mutations more rapidly compared to the first and second positions. Consequently, third codon positions are deemed unreliable for reconstructing phylogenetic relationships within the four tribes. Furthermore, sequence divergence in the outgroup *Chironomus* species was markedly less than that observed in the ingroup taxa.

**FIGURE 7:**
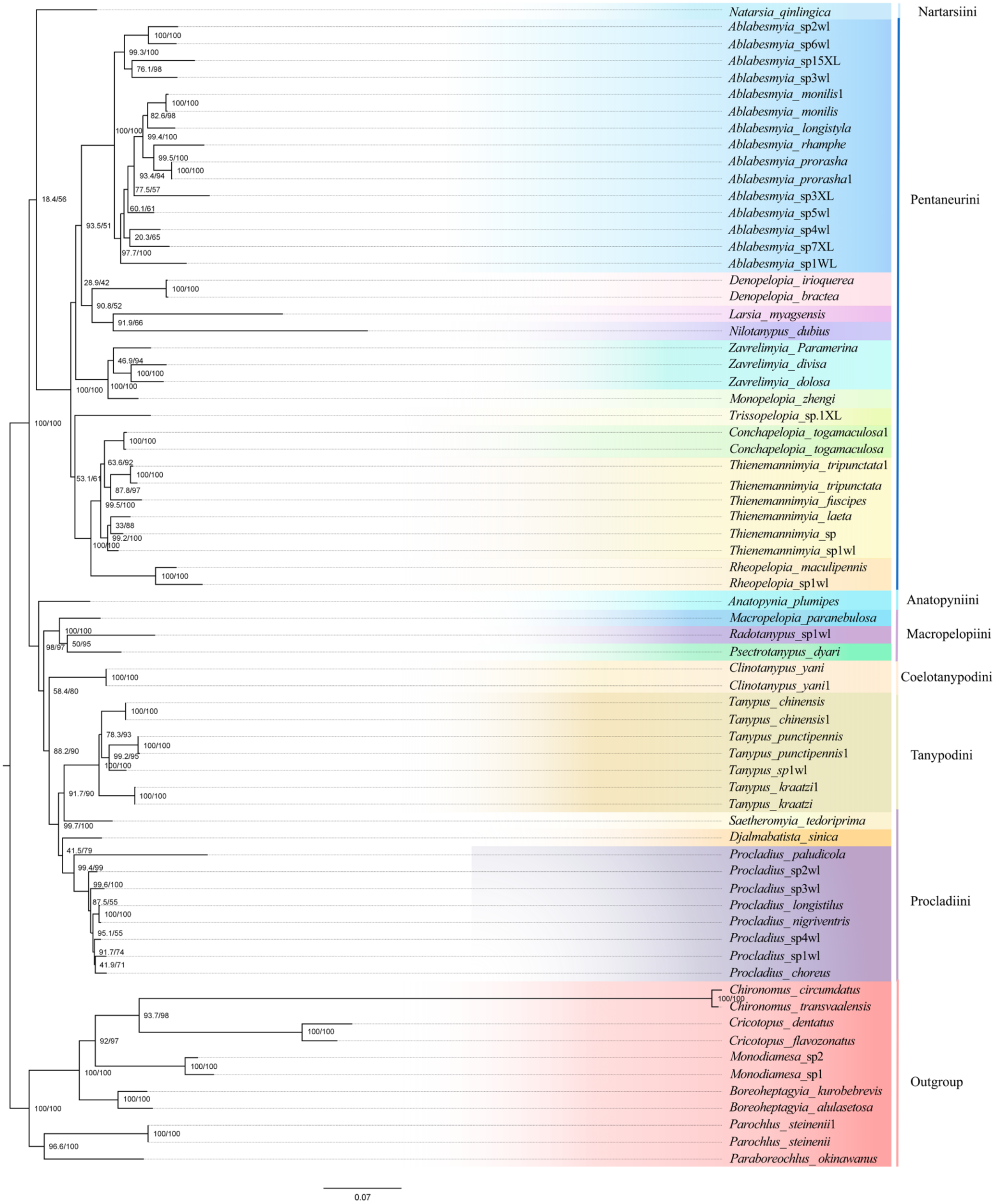
Phylogenetic ML tree of the genus Tanypondinae, based on analysis PCG12_rRNA in partition.

Phylogenetic inference, performed with BI and ML across the 10 datasets, consistently yielded concordant tree architectures, yet disparities appeared in branching configurations and nodal support indices (Figure 7 and Figures S1–S8). Phylogenetic inference resolves the 50 Tanypodinae species into seven discrete lineages that correspond precisely to the seven recognized tribes: Pentaneurini, Procladiini, Tanypodini, Coelotanypodini, Nartarsiini, Anatopynini, and Macropelopiini, following Silva and Ekrem (2016), Krosch et al. (2017, 2021) and Xiao et al. (2025).

The mitochondrial phylogenomic analysis strongly supports the monophyly of Pentaneurini and its sister‐group relationship to (Coelotanypodini + (Macropelopiini + (Tanypodini + Procladiini))), aligning with prior studies (Xiao et al. 2025). The Tanypodini‐Procladiini sister relationship is also strongly supported, echoing Silva and Ekrem's (2016) morphological findings. Within Pentaneurini, the monophyly of *Ablabesmyia* is confirmed (Liu et al. 2025), and the topology shows (*Ablabesmyia* + *Nilotanypus*) as sister to (*Rheopelopia* + (*Thienemannimyia* + *Conchapelopia*) + (*Zarelimyia* + Denopelopia)), which mirrors the global dataset analysis of Tanypodinae using *COI*, *28S*, and *CAD*, bolstering confidence in future genomic phylogenetic research (Krosch et al. 2017, 2021). This discovery elucidates key evolutionary dynamics within Tanypodinae, underscoring the imperative for expanded taxonomic sampling to resolve the subfamily's intricate phylogenetic relationships.

In the study by Silva, Anatopyniini and the clade comprising Fittkauimyiini + Macropelopiini were recovered as sister groups. However, due to the absence of Fittkauimyiini samples in our dataset, our study could only confirm the sister‐group relationship between Anatopyniini and Macropelopiini. Notably, both our study and Silva consistently support the monophyly of Nartarsiini, identifying it as a distinct lineage that forms a sister group with Pentaneurinir (Table S2).

## 4 Conclusions

This study presents the first fully annotated and assembled mitochondrial genomes for twenty‐three species spanning all five tribes of the subfamily Tanypodinae (Diptera: Chironomidae). These novel mitogenomes exhibit conserved architectural characteristics and base composition consistent with existing records for Chironomidae, substantially enlarging the available genomic resources for this family.

Despite pronounced morphological differentiation across larval, pupal, and adult life stages within Chironomidae, phylogenetic hypotheses inferred from morphology, limited gene markers, and complete mitogenomes frequently demonstrate significant incongruence. Molecular analyses, however, continue to affirm the enduring relevance of detailed morphological examination in chironomid systematics. While comprehensive mitogenomic datasets offer considerable potential for resolving evolutionary relationships, their interpretation necessitates critical evaluation. A robust phylogenetic framework must therefore synthesize developmental morphology, biogeography, and life‐history traits with genomic evidence to elucidate the group's intrinsic evolutionary pathways.

## Author Contributions

**Wen‐Bin Liu:** conceptualization (equal), resources (equal), software (equal), writing – original draft (equal). **Jia‐Xin Nie:** formal analysis (equal), methodology (equal), validation (equal). **Ya‐Ning Tang:** data curation (equal), investigation (equal), software (equal). **Zi‐Ming Shao:** data curation (equal), formal analysis (equal), resources (equal). **Cheng‐Yan Wang:** data curation (equal), resources (equal), visualization (equal). **Chun‐Cai Yan:** project administration (equal), supervision (equal), writing – review and editing (equal).

## Conflicts of Interest

The authors declare no conflicts of interest.

## Supporting information


**Figure S1:** Phylogenetic BI tree of the genus Tanypondinae, based on PCG_faa.
**Figure S2:** Phylogenetic ML tree of the genus Tanypondinae, based on PCG_faa in partition.
**Figure S3:** Phylogenetic ML tree of the genus Tanypondinae, based on PCG_fna in partition.
**Figure S4:** Phylogenetic BI tree of the genus Tanypondinae, based on PCG_rRNA.
**Figure S5:** Phylogenetic ML tree of the genus Tanypondinae, based on PCG_rRNA in partition.
**Figure S6:** Phylogenetic BI tree of the genus Tanypondinae, based on PCG_12rRNA.
**Figure S7:** Phylogenetic BI tree of the genus Tanypondinae, based on PCG_12rRNA.
**Figure S8:** Phylogenetic ML tree of the genus Tanypondinae, based on PCG_12fna in partition.
**Table S1:** Nucleotide composition of 38 mitogenomes.

## Data Availability

Regarding sequence accessibility, the newly assembled mitogenomes have been deposited in the GenBank repository under NCBI, with accession identifiers detailed in Table 2; all requisite datasets are additionally provided as Supporting files.
