# Supplementary figures and images for: Phylogenetic Analysis of Tanypodinae (Chironomidae, Diptera) Infered From Whole Mitochondrial Genomes
Source: Ecol Evol. 2026 Feb 13;16(2):e72975. doi: 10.1002/ece3.72975 (PMC12905465; doi:10.1002/ece3.72975)

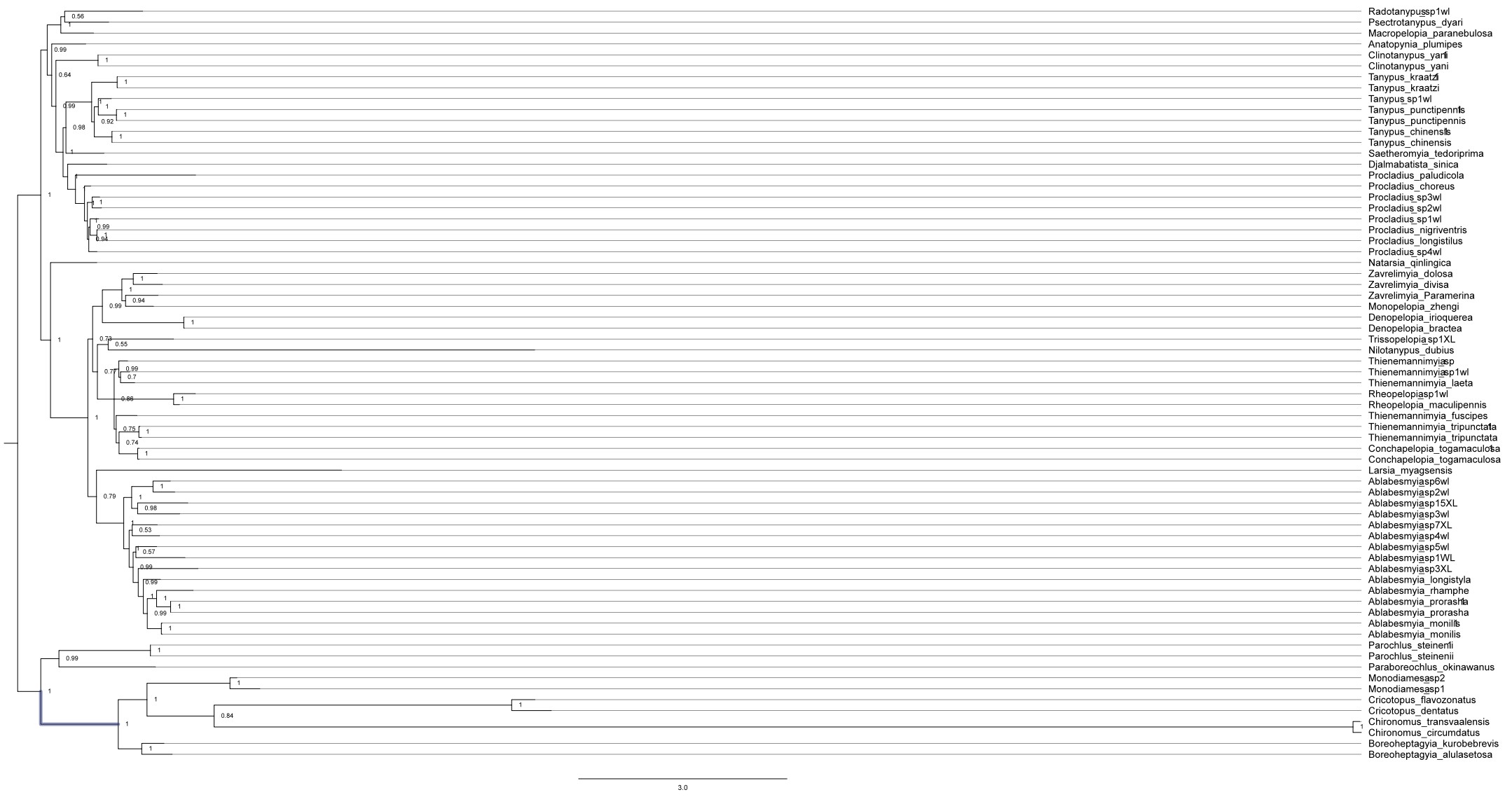

Supplement: Supplementary file 1 — Figure S1: Phylogenetic BI tree of the genus Tanypondinae, based on PCG_faa. Figure S2: Phylogenetic ML tree of the genus Tanypondinae, based on PCG_faa in partition. Figure S3: Phylogenetic ML tree of the genus Tanypondinae, based on PCG_fna in partition. Figure S4: Phylogenetic BI tree of the genus Tanypondinae, based on PCG_rRNA. Figure S5: Phylogenetic ML tree of the genus Tanypondinae, based on PCG_rRNA in partition. Figure S6: Phylogenetic BI tree of the genus Tanypondinae, based on PCG_12rRNA. Figure S7: Phylogenetic BI tree of the genus Tanypondinae, based on PCG_12rRNA. Figure S8: Phylogenetic ML tree of the genus Tanypondinae, based on PCG_12fna in partition. Table S1: Nucleotide composition of 38 mitogenomes. [file ECE3-16-e72975-s001.zip › ece372975-sup-0001-FigureS1.jpg]

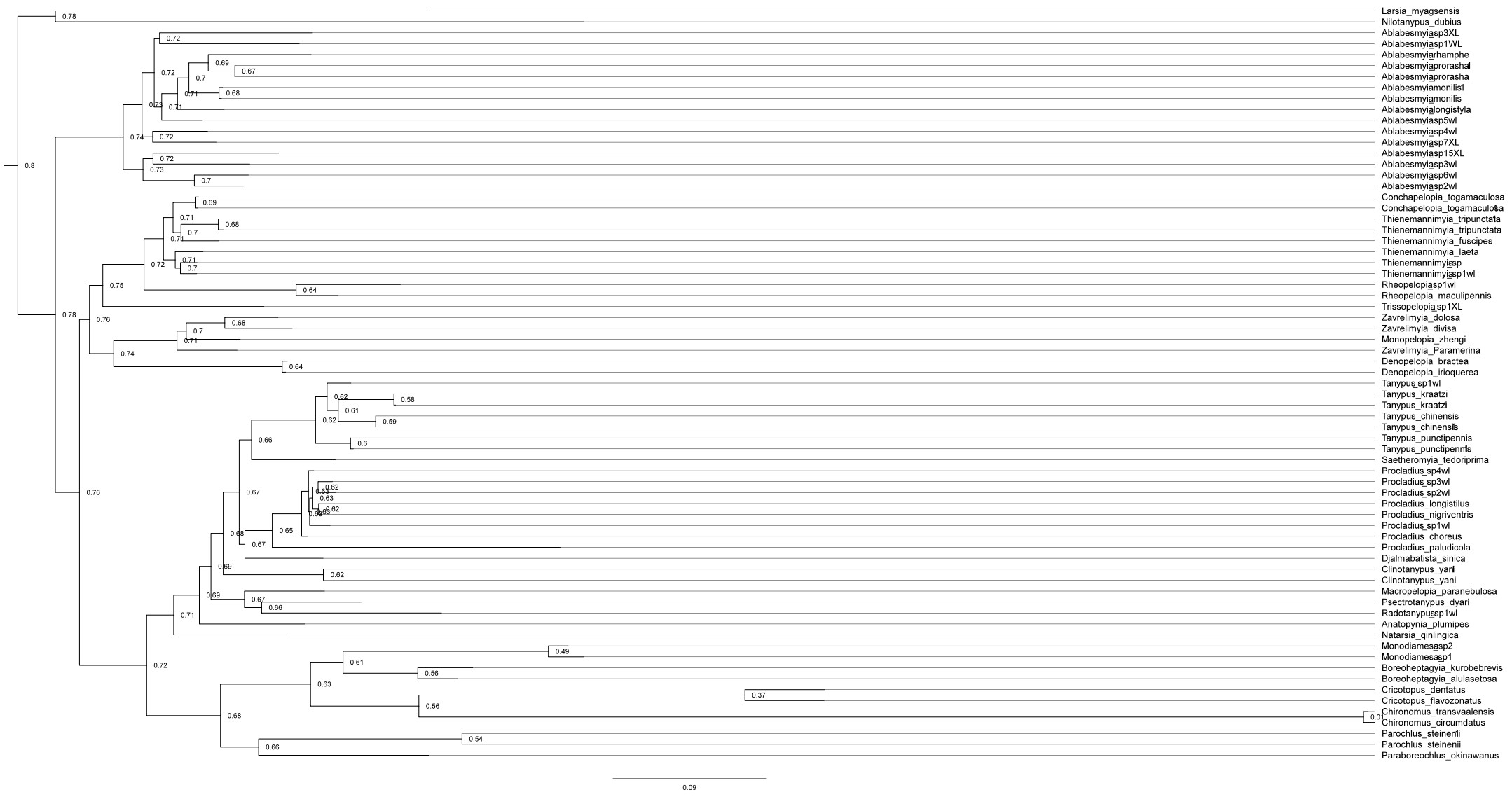

Supplement: Supplementary file 1 — Figure S1: Phylogenetic BI tree of the genus Tanypondinae, based on PCG_faa. Figure S2: Phylogenetic ML tree of the genus Tanypondinae, based on PCG_faa in partition. Figure S3: Phylogenetic ML tree of the genus Tanypondinae, based on PCG_fna in partition. Figure S4: Phylogenetic BI tree of the genus Tanypondinae, based on PCG_rRNA. Figure S5: Phylogenetic ML tree of the genus Tanypondinae, based on PCG_rRNA in partition. Figure S6: Phylogenetic BI tree of the genus Tanypondinae, based on PCG_12rRNA. Figure S7: Phylogenetic BI tree of the genus Tanypondinae, based on PCG_12rRNA. Figure S8: Phylogenetic ML tree of the genus Tanypondinae, based on PCG_12fna in partition. Table S1: Nucleotide composition of 38 mitogenomes. [file ECE3-16-e72975-s001.zip › ece372975-sup-0002-FigureS2.jpg]

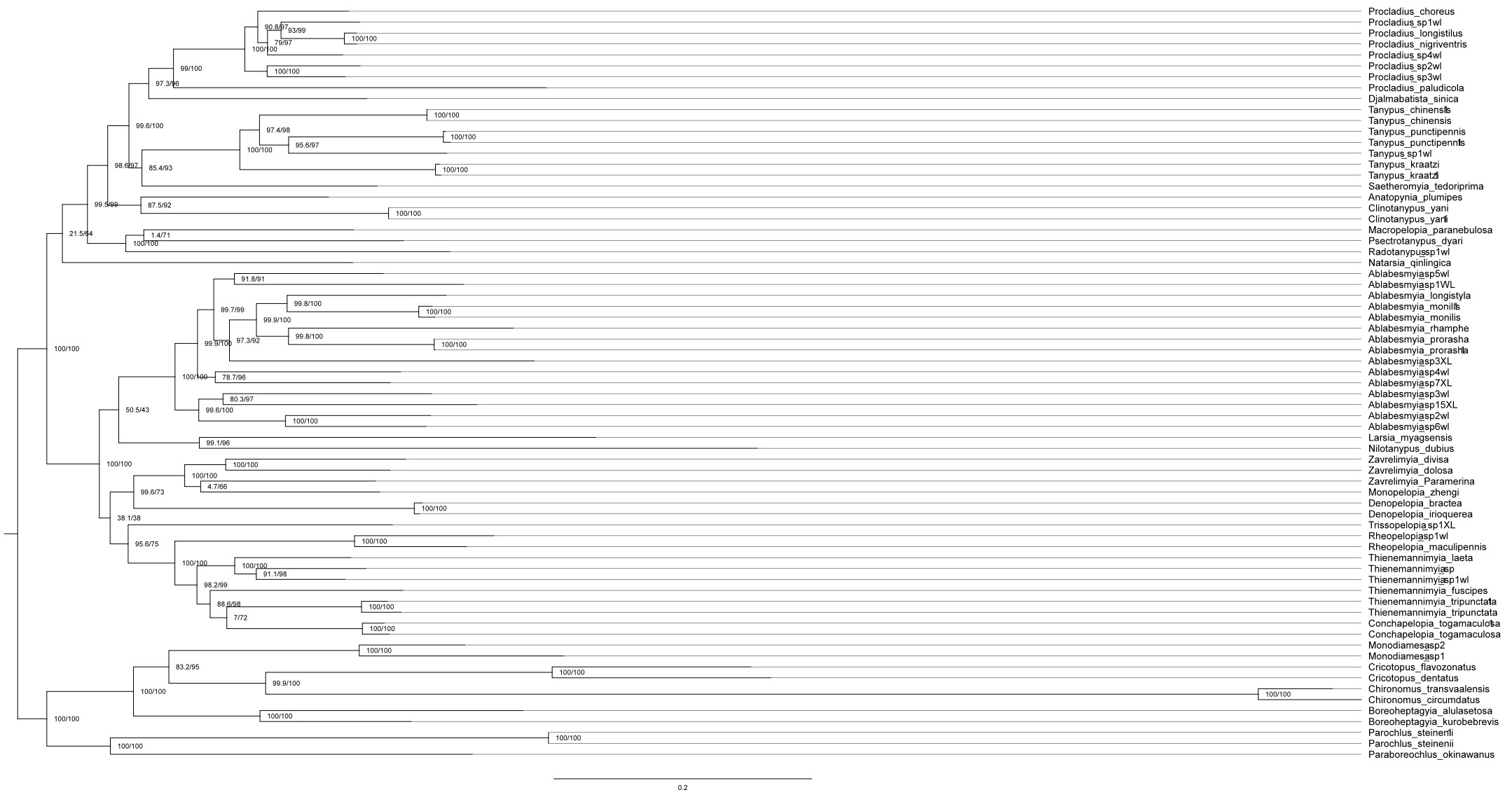

Supplement: Supplementary file 1 — Figure S1: Phylogenetic BI tree of the genus Tanypondinae, based on PCG_faa. Figure S2: Phylogenetic ML tree of the genus Tanypondinae, based on PCG_faa in partition. Figure S3: Phylogenetic ML tree of the genus Tanypondinae, based on PCG_fna in partition. Figure S4: Phylogenetic BI tree of the genus Tanypondinae, based on PCG_rRNA. Figure S5: Phylogenetic ML tree of the genus Tanypondinae, based on PCG_rRNA in partition. Figure S6: Phylogenetic BI tree of the genus Tanypondinae, based on PCG_12rRNA. Figure S7: Phylogenetic BI tree of the genus Tanypondinae, based on PCG_12rRNA. Figure S8: Phylogenetic ML tree of the genus Tanypondinae, based on PCG_12fna in partition. Table S1: Nucleotide composition of 38 mitogenomes. [file ECE3-16-e72975-s001.zip › ece372975-sup-0003-FigureS3.jpg]

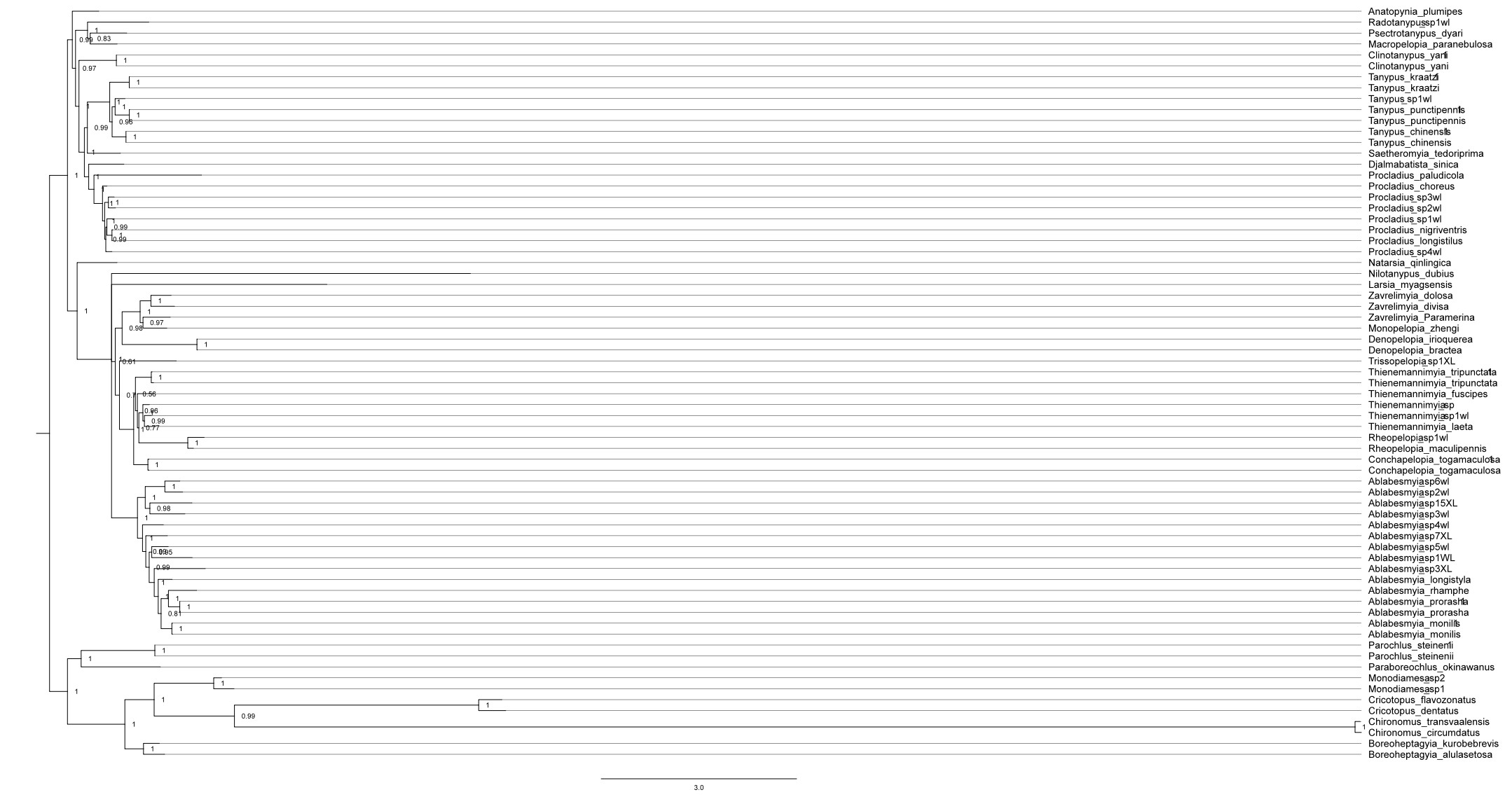

Supplement: Supplementary file 1 — Figure S1: Phylogenetic BI tree of the genus Tanypondinae, based on PCG_faa. Figure S2: Phylogenetic ML tree of the genus Tanypondinae, based on PCG_faa in partition. Figure S3: Phylogenetic ML tree of the genus Tanypondinae, based on PCG_fna in partition. Figure S4: Phylogenetic BI tree of the genus Tanypondinae, based on PCG_rRNA. Figure S5: Phylogenetic ML tree of the genus Tanypondinae, based on PCG_rRNA in partition. Figure S6: Phylogenetic BI tree of the genus Tanypondinae, based on PCG_12rRNA. Figure S7: Phylogenetic BI tree of the genus Tanypondinae, based on PCG_12rRNA. Figure S8: Phylogenetic ML tree of the genus Tanypondinae, based on PCG_12fna in partition. Table S1: Nucleotide composition of 38 mitogenomes. [file ECE3-16-e72975-s001.zip › ece372975-sup-0004-FigureS4.jpg]

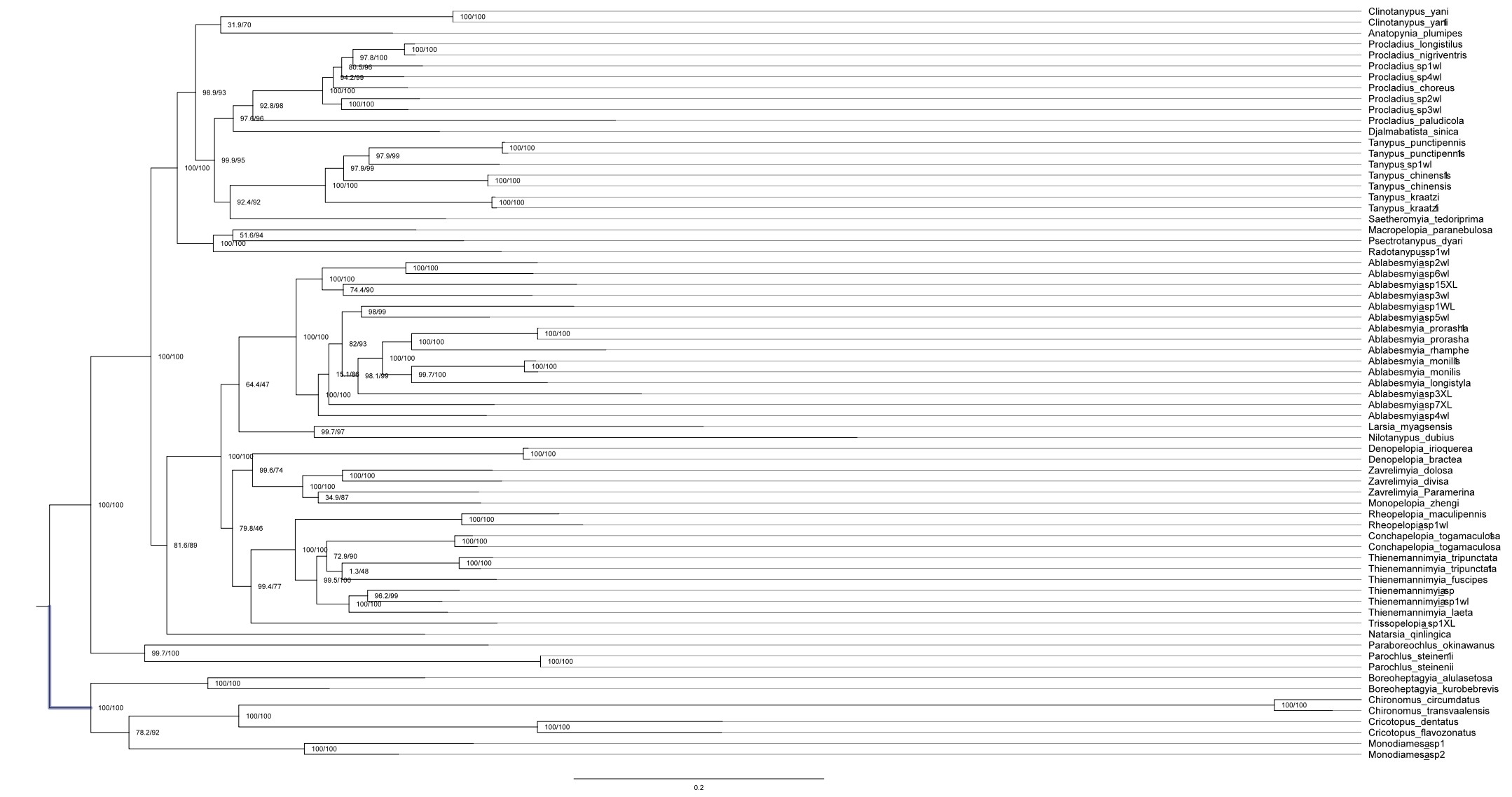

Supplement: Supplementary file 1 — Figure S1: Phylogenetic BI tree of the genus Tanypondinae, based on PCG_faa. Figure S2: Phylogenetic ML tree of the genus Tanypondinae, based on PCG_faa in partition. Figure S3: Phylogenetic ML tree of the genus Tanypondinae, based on PCG_fna in partition. Figure S4: Phylogenetic BI tree of the genus Tanypondinae, based on PCG_rRNA. Figure S5: Phylogenetic ML tree of the genus Tanypondinae, based on PCG_rRNA in partition. Figure S6: Phylogenetic BI tree of the genus Tanypondinae, based on PCG_12rRNA. Figure S7: Phylogenetic BI tree of the genus Tanypondinae, based on PCG_12rRNA. Figure S8: Phylogenetic ML tree of the genus Tanypondinae, based on PCG_12fna in partition. Table S1: Nucleotide composition of 38 mitogenomes. [file ECE3-16-e72975-s001.zip › ece372975-sup-0005-FigureS5.jpg]

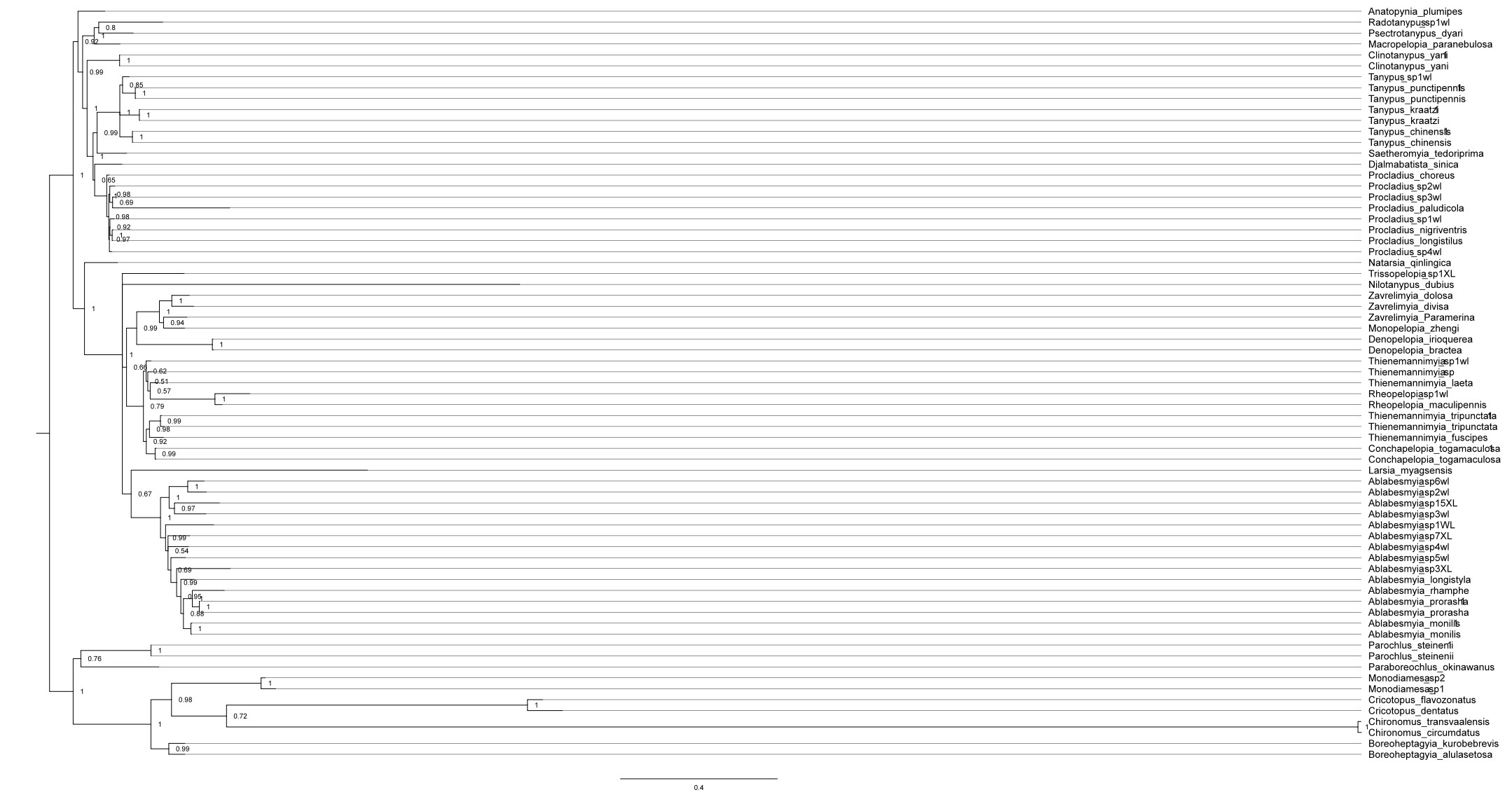

Supplement: Supplementary file 1 — Figure S1: Phylogenetic BI tree of the genus Tanypondinae, based on PCG_faa. Figure S2: Phylogenetic ML tree of the genus Tanypondinae, based on PCG_faa in partition. Figure S3: Phylogenetic ML tree of the genus Tanypondinae, based on PCG_fna in partition. Figure S4: Phylogenetic BI tree of the genus Tanypondinae, based on PCG_rRNA. Figure S5: Phylogenetic ML tree of the genus Tanypondinae, based on PCG_rRNA in partition. Figure S6: Phylogenetic BI tree of the genus Tanypondinae, based on PCG_12rRNA. Figure S7: Phylogenetic BI tree of the genus Tanypondinae, based on PCG_12rRNA. Figure S8: Phylogenetic ML tree of the genus Tanypondinae, based on PCG_12fna in partition. Table S1: Nucleotide composition of 38 mitogenomes. [file ECE3-16-e72975-s001.zip › ece372975-sup-0006-FigureS6.jpg]

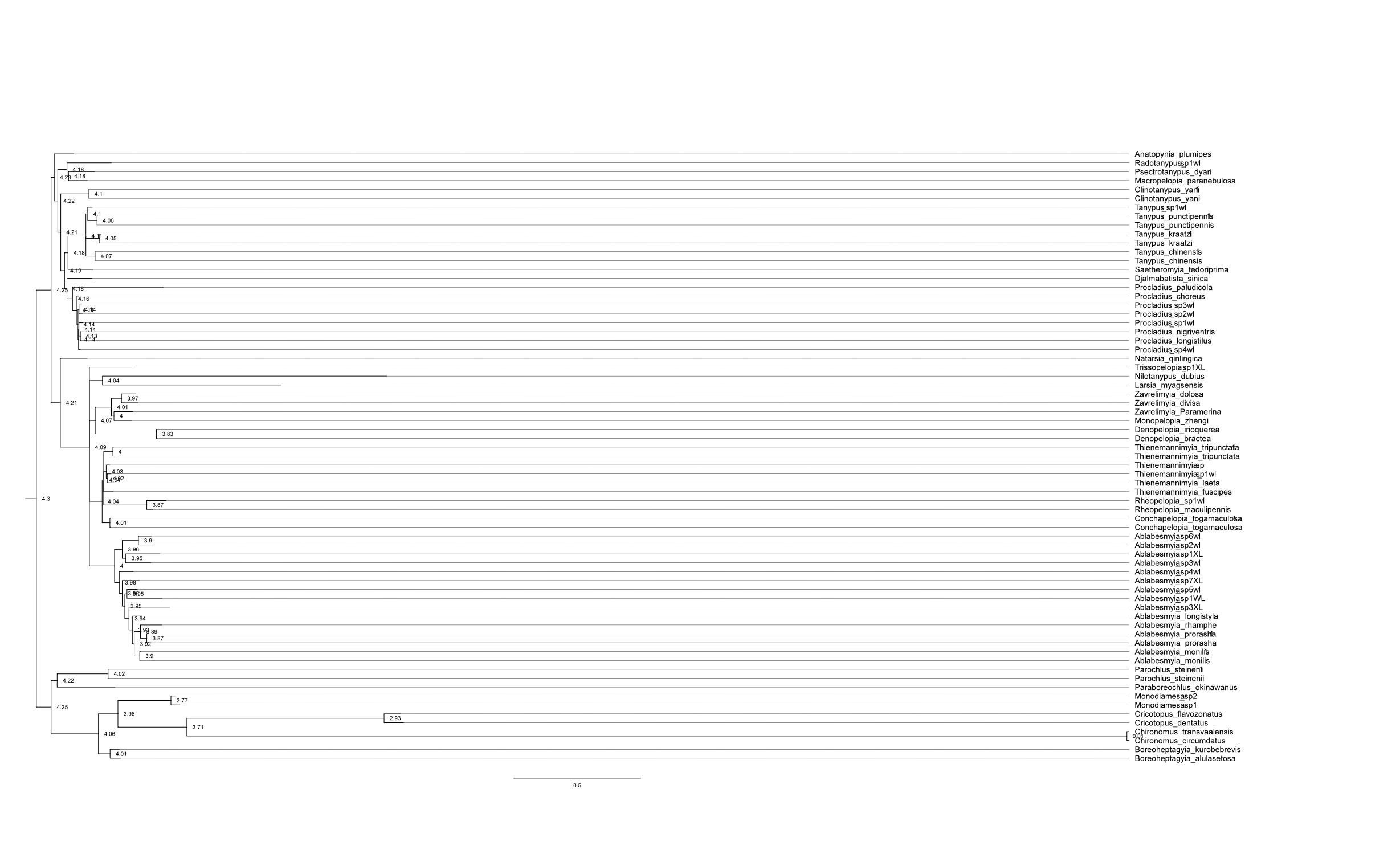

Supplement: Supplementary file 1 — Figure S1: Phylogenetic BI tree of the genus Tanypondinae, based on PCG_faa. Figure S2: Phylogenetic ML tree of the genus Tanypondinae, based on PCG_faa in partition. Figure S3: Phylogenetic ML tree of the genus Tanypondinae, based on PCG_fna in partition. Figure S4: Phylogenetic BI tree of the genus Tanypondinae, based on PCG_rRNA. Figure S5: Phylogenetic ML tree of the genus Tanypondinae, based on PCG_rRNA in partition. Figure S6: Phylogenetic BI tree of the genus Tanypondinae, based on PCG_12rRNA. Figure S7: Phylogenetic BI tree of the genus Tanypondinae, based on PCG_12rRNA. Figure S8: Phylogenetic ML tree of the genus Tanypondinae, based on PCG_12fna in partition. Table S1: Nucleotide composition of 38 mitogenomes. [file ECE3-16-e72975-s001.zip › ece372975-sup-0007-FigureS7.jpg]

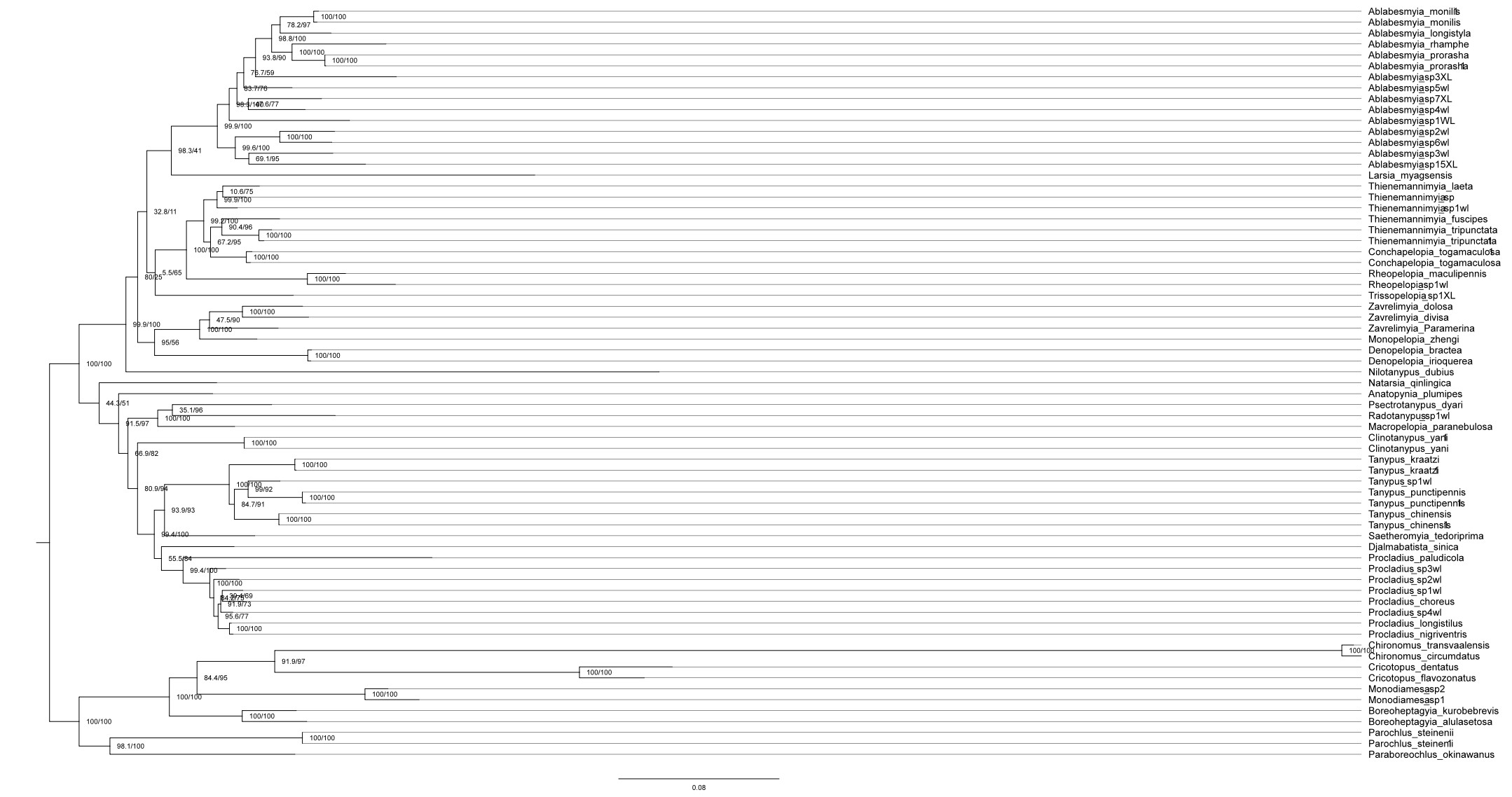

Supplement: Supplementary file 1 — Figure S1: Phylogenetic BI tree of the genus Tanypondinae, based on PCG_faa. Figure S2: Phylogenetic ML tree of the genus Tanypondinae, based on PCG_faa in partition. Figure S3: Phylogenetic ML tree of the genus Tanypondinae, based on PCG_fna in partition. Figure S4: Phylogenetic BI tree of the genus Tanypondinae, based on PCG_rRNA. Figure S5: Phylogenetic ML tree of the genus Tanypondinae, based on PCG_rRNA in partition. Figure S6: Phylogenetic BI tree of the genus Tanypondinae, based on PCG_12rRNA. Figure S7: Phylogenetic BI tree of the genus Tanypondinae, based on PCG_12rRNA. Figure S8: Phylogenetic ML tree of the genus Tanypondinae, based on PCG_12fna in partition. Table S1: Nucleotide composition of 38 mitogenomes. [file ECE3-16-e72975-s001.zip › ece372975-sup-0008-FigureS8.jpg]
